# (2*S*,4′*R*,5′*R*)-(*E*)-*tert*-Butyl 2-acetyl-2-(2-oxo-5-phenyl-1,3-dioxolan-4-ylmeth­yl)-5-phenyl­pent-4-enoate

**DOI:** 10.1107/S1600536808002651

**Published:** 2008-01-30

**Authors:** David J. Fox, Daniel Sejer Pedersen, Stuart Warren

**Affiliations:** a Department of Chemistry, University of Cambridge, Lensfield Road, Cambridge CB2 1EW, England

## Abstract

The title compound, C_27_H_30_O_6_, was prepared by monodihydroxy­lation of the bis-olefin (*E*,*E*)-*tert*-butyl 2-acetyl-2-cinnamyl-5-phenyl­pent-4-enoate using standard Sharpless asymmetric dihydroxy­lation conditions, followed by treatment with 1,1′-carbonyl diimidazole. In the crystal structure, the phenyl rings form an intra­molecular edge-to-face C—H⋯π contact with an inter­planar angle of 56.4° and a H⋯centroid distance of 3.03 Å.

## Related literature

For related literature, see: Fox *et al.* (2006 ▶); Kolb *et al.* (1994 ▶).
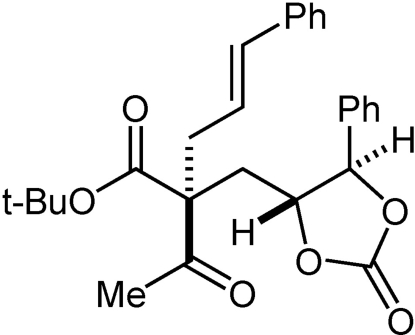


## Experimental

### 

#### Crystal data

C_27_H_30_O_6_
*M* *_r_* = 450.51
Orthorhombic, 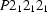

*a* = 6.4707 (2) Å
*b* = 7.7258 (4) Å
*c* = 49.803 (3) Å
*V* = 2489.7 (2) Å^3^
*Z* = 4
Mo *K*α radiation
μ = 0.08 mm^−1^
*T* = 200 (2) K
0.37 × 0.25 × 0.05 mm

#### Data collection

Nonius KappaCCD diffractometer
Absorption correction: multi-scan (*SORTAV*; Blessing, 1995 ▶) *T*_min_ = 0.817, *T*_max_ = 0.996
4524 measured reflections
1806 independent reflections
1188 reflections with *I* > 2σ(*I*)
*R*_int_ = 0.070

#### Refinement

*R*[*F* ^2^ > 2σ(*F* ^2^)] = 0.080
*wR*(*F* ^2^) = 0.256
*S* = 1.10
1806 reflections
274 parameters
1 restraint
H-atom parameters constrained
Δρ_max_ = 0.64 e Å^−3^
Δρ_min_ = −0.68 e Å^−3^

### 

Data collection: *COLLECT* (Nonius, 1998 ▶); cell refinement: *SCALEPACK* (Otwinowski & Minor, 1997 ▶); data reduction: *DENZO* (Otwinowski & Minor, 1997 ▶) and *SCALEPACK*; program(s) used to solve structure: *SIR92* (Altomare *et al.*, 1994 ▶); program(s) used to refine structure: *SHELXL97* (Sheldrick, 2008 ▶); molecular graphics: *SHELXTL* (Sheldrick, 2008 ▶); software used to prepare material for publication: *SHELXTL*.

## Supplementary Material

Crystal structure: contains datablocks global, I. DOI: 10.1107/S1600536808002651/hg2374sup1.cif

Structure factors: contains datablocks I. DOI: 10.1107/S1600536808002651/hg2374Isup2.hkl

Additional supplementary materials:  crystallographic information; 3D view; checkCIF report

## Figures and Tables

**Table 1:** Hydrogen-bond geometry (Å, °) *Cg* is the centroid of the C7–C12 ring.

*D*—H⋯*A*	*D*—H	H⋯*A*	*D*⋯*A*	*D*—H⋯*A*
C19—H19*A*⋯*Cg*	0.95	3.03	3.757	135

**Fig. 1.**
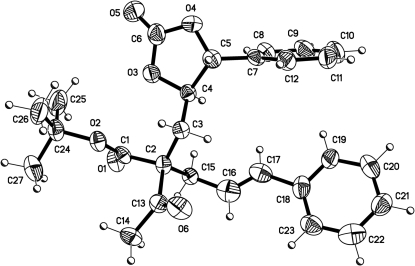
Molecular structure with displacement parameters drawn at the 30% probability level for non-H atoms.

**Fig. 2.**
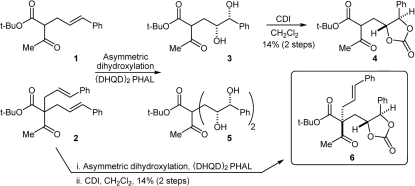
Summary of the synthetic procedure. CDI = 1,1'-carbonyldiimidazole, (DHQD)2PHAL = Hydroquinidine 1,4-phthalazinediyl diether

## supplementary crystallographic information

### Comment

Recently, we published a method for the synthesis of dihydrofurans containing a diphenylphosphinoyl group by intramolecular ring opening of cyclic carbonates (Fox *et al.*, 2006). We are currently seeking to extend this methodology with other anion-stabilizing groups. In particular, we are interested in replacing the diphenylphosphinoyl group with a carboxylic ester. When we exposed (*E*,*E*)-*tert*-butyl 2-acetyl-2-cinnamyl-5-phenylpent-4-enoate to the standard Sharpless asymmetric dihydroxylation conditions (Kolb *et al.*, 1994), followed by treatment with 1,1'-carbonyl diimidazole we obtained a significant amount (20%) of the title compound where only one olefin had been dihydroxylated.

### Experimental

The synthetic procedure is summarized in Fig. 2. By a method analogous to that reported by Sharpless and co-workers (Kolb *et al.*, 1994), *tert*-butyl ester 1 (3.0 g, 10.9 mmol; 5:1 mixture of 1 and 2) was dissolved in *t*-BuOH (100 ml) to give a clear solution. Water (100 ml) was added and the mixture was cooled to 278 K. A freshly made mixture of K_2_OsO_4_.2H_2_O (1 mol %), K_3_Fe(CN)_6_ (3 equiv.), K_2_CO_3_ (3 equiv.), MeSO_2_NH_2_ (1 equiv.) and hydroquinidine 1,4-phthalazinediyl diether (denoted (DHQD)_2_PHAL, 2 mol %) was added to the cooled solution in one portion and it was stirred vigorously for 24 h. Sodium sulfite (*ca* 10 equiv.) was added and the reaction allowed to warm to room temperature with vigorous stirring. The slurry was transferred to a separatory funnel with water (200 ml) and extracted with ethyl acetate (3 × 100 ml). The combined organic extracts were washed with aqueous sulfate buffer (100 ml), saturated aqueous NaHCO_3_ (100 ml), dried (Na_2_SO_4_), filtered and evaporated under reduced pressure. The residue was dissolved in dichloromethane (100 ml) and 1,1'-carbonyldiimidazole (1.5 equiv.) was added to the stirred solution at room temperature. The reaction mixture was stirred until completion to give a complex mixture of products. Water (100 ml) was added and the mixture transferred to a separatory funnel with brine (100 ml) and extracted with dichloromethane (3 × 100 ml). The combined organic phases were dried (Na_2_SO_4_), filtered and the solvent removed *in vacuo* to give the crude product that was purified through a combination of crystallizations and column chromatography to give 4 (338 mg, 14%) as a clear gum (1:1 mixture of diastereoisomers) and the title compound (denoted 6 in Fig. 2, 112 mg, 14%) as colourless plates (a single diastereoisomer). m.p. (EtOAc, pentane) = 449–450 K.

### Refinement

H atoms were placed geometrically and allowed to ride during refinement with C—H = 0.95–1.00 Å and with *U*_iso_(H) = 1.2 or 1.5*U*_eq_(C). A combination of relatively thin plates and large unit-cell volume gave rise to relatively weak diffraction. The resulting structure is therefore of low precision. Although the molecular geometry was reasonable when unconstrained, the phenyl rings were constrained to be regular hexagons in an effort to improve the data-to-parameter ratio. One restraint was necessary: the C16=C17 bond was restrained to 1.35 (1) Å. In the absence of significant anomalous scattering effects, 770 Friedel pairs were merged as equivalent data. The absolute structure is based on the known stereochemical outcome of the asymmetric dihydroxylation.

### Crystal data

**Table:**

C_27_H_30_O_6_	*F*_000_ = 960
*M**_r_* = 450.51	*D*_x_ = 1.202 Mg m^−^^3^
Orthorhombic, *P*2_1_2_1_2_1_	Mo *K*α radiation λ = 0.71073 Å
Hall symbol: P 2ac 2ab	Cell parameters from 21008 reflections
*a* = 6.4707 (2) Å	θ = 1.0–25.0º
*b* = 7.7258 (4) Å	µ = 0.08 mm^−^^1^
*c* = 49.803 (3) Å	*T* = 200 (2) K
*V* = 2489.7 (2) Å^3^	Plate, colourless
*Z* = 4	0.37 × 0.25 × 0.05 mm

### Data collection

**Table:**

Nonius KappaCCD diffractometer	1806 independent reflections
Radiation source: fine-focus sealed tube	1188 reflections with *I* > 2σ(*I*)
Monochromator: graphite	*R*_int_ = 0.070
*T* = 200(2) K	θ_max_ = 24.9º
ω and φ scans	θ_min_ = 3.6º
Absorption correction: multi-scan(SORTAV; Blessing, 1995)	*h* = −7→7
*T*_min_ = 0.817, *T*_max_ = 0.996	*k* = −9→9
4524 measured reflections	*l* = −58→58

### Refinement

**Table:**

Refinement on *F*^2^	Secondary atom site location: difference Fourier map
Least-squares matrix: full	Hydrogen site location: inferred from neighbouring sites
*R*[*F*^2^ > 2σ(*F*^2^)] = 0.080	H-atom parameters constrained
*wR*(*F*^2^) = 0.256	*w* = 1/[σ^2^(*F*_o_^2^) + (0.175*P*)^2^] where *P* = (*F*_o_^2^ + 2*F*_c_^2^)/3
*S* = 1.10	(Δ/σ)_max_ < 0.001
1806 reflections	Δρ_max_ = 0.64 e Å^−^^3^
274 parameters	Δρ_min_ = −0.68 e Å^−^^3^
1 restraint	Extinction correction: none
Primary atom site location: structure-invariant direct methods	Absolute structure: In the absence of significant anomalous scattering effects, 770 Friedel pairs have been merged as equivalent data.

### Special details

**Table:**

Geometry. All e.s.d.'s (except the e.s.d. in the dihedral angle between two l.s. planes) are estimated using the full covariance matrix. The cell e.s.d.'s are taken into account individually in the estimation of e.s.d.'s in distances, angles and torsion angles; correlations between e.s.d.'s in cell parameters are only used when they are defined by crystal symmetry. An approximate (isotropic) treatment of cell e.s.d.'s is used for estimating e.s.d.'s involving l.s. planes.
Refinement. Refinement of *F*^2^ against ALL reflections. The weighted *R*-factor *wR* and goodness of fit *S* are based on *F*^2^, conventional *R*-factors *R* are based on *F*, with *F* set to zero for negative *F*^2^. The threshold expression of *F*^2^ > σ(*F*^2^) is used only for calculating *R*-factors(gt) *etc*. and is not relevant to the choice of reflections for refinement. *R*-factors based on *F*^2^ are statistically about twice as large as those based on *F*, and *R*- factors based on ALL data will be even larger.

### Fractional atomic coordinates and isotropic or equivalent isotropic displacement parameters (Å^2^)

**Table:**

	*x*	*y*	*z*	*U*_iso_*/*U*_eq_	
O1	0.5651 (9)	0.7809 (10)	0.79920 (11)	0.078 (2)	
O2	0.7465 (8)	0.5341 (8)	0.80759 (10)	0.0608 (15)	
O3	0.4141 (8)	0.4621 (9)	0.84707 (10)	0.0691 (17)	
O4	0.3075 (9)	0.3331 (8)	0.88419 (13)	0.0799 (19)	
O5	0.1713 (12)	0.2537 (12)	0.84465 (15)	0.118 (3)	
O6	1.1049 (8)	0.7479 (10)	0.85656 (13)	0.086 (2)	
C1	0.6727 (11)	0.6903 (13)	0.81319 (16)	0.057 (2)	
C2	0.7525 (10)	0.7483 (12)	0.84096 (14)	0.053 (2)	
C3	0.7220 (10)	0.6075 (11)	0.86277 (15)	0.050 (2)	
H3A	0.7955	0.5014	0.8570	0.060*	
H3B	0.7876	0.6480	0.8796	0.060*	
C4	0.5051 (11)	0.5618 (12)	0.86863 (14)	0.053 (2)	
H4A	0.4234	0.6704	0.8712	0.063*	
C5	0.4734 (13)	0.4430 (12)	0.89308 (14)	0.062 (2)	
H5A	0.6005	0.3717	0.8960	0.074*	
C6	0.2887 (15)	0.3408 (16)	0.8573 (2)	0.082 (3)	
C7	0.4154 (9)	0.5309 (9)	0.91885 (9)	0.058 (2)	
C8	0.2322 (8)	0.6241 (9)	0.92076 (11)	0.072 (3)	
H8A	0.1450	0.6355	0.9055	0.086*	
C9	0.1766 (10)	0.7005 (9)	0.94498 (15)	0.090 (3)	
H9A	0.0515	0.7642	0.9463	0.108*	
C10	0.3042 (13)	0.6837 (9)	0.96728 (11)	0.101 (4)	
H10A	0.2662	0.7360	0.9838	0.121*	
C11	0.4874 (12)	0.5905 (10)	0.96537 (9)	0.096 (3)	
H11A	0.5746	0.5791	0.9806	0.116*	
C12	0.5429 (9)	0.5141 (9)	0.94116 (12)	0.075 (3)	
H12A	0.6681	0.4504	0.9399	0.090*	
C13	0.9897 (13)	0.7829 (13)	0.83825 (17)	0.064 (2)	
C14	1.0678 (12)	0.8710 (13)	0.81315 (16)	0.073 (3)	
H14A	1.2177	0.8868	0.8144	0.110*	
H14B	1.0007	0.9840	0.8113	0.110*	
H14C	1.0356	0.7992	0.7975	0.110*	
C15	0.6514 (11)	0.9228 (11)	0.84840 (15)	0.055 (2)	
H15A	0.6708	1.0055	0.8334	0.066*	
H15B	0.5011	0.9055	0.8509	0.066*	
C16	0.7411 (16)	0.9975 (13)	0.8733 (2)	0.094 (4)	
H16A	0.8774	1.0426	0.8722	0.112*	
C17	0.6501 (16)	1.0065 (14)	0.8962 (2)	0.093 (3)	
H17A	0.5094	0.9719	0.8971	0.111*	
C18	0.7538 (12)	1.0696 (10)	0.92249 (10)	0.083 (3)	
C19	0.6353 (9)	1.0352 (9)	0.94515 (13)	0.078 (3)	
H19A	0.5048	0.9801	0.9434	0.094*	
C20	0.7078 (11)	1.0815 (10)	0.97042 (10)	0.092 (3)	
H20A	0.6268	1.0580	0.9859	0.110*	
C21	0.8987 (12)	1.1622 (10)	0.97304 (13)	0.091 (3)	
H21A	0.9483	1.1938	0.9903	0.109*	
C22	1.0172 (9)	1.1966 (9)	0.95038 (19)	0.099 (3)	
H22A	1.1478	1.2517	0.9522	0.119*	
C23	0.9448 (11)	1.1503 (10)	0.92511 (15)	0.090 (3)	
H23A	1.0258	1.1738	0.9096	0.109*	
C24	0.6977 (13)	0.4463 (15)	0.78174 (16)	0.073 (3)	
C25	0.4660 (18)	0.445 (2)	0.7773 (2)	0.118 (5)	
H25A	0.4169	0.5632	0.7745	0.176*	
H25B	0.3977	0.3952	0.7932	0.176*	
H25C	0.4335	0.3742	0.7615	0.176*	
C26	0.796 (2)	0.2715 (13)	0.7865 (2)	0.110 (4)	
H26A	0.7212	0.2104	0.8007	0.165*	
H26B	0.9407	0.2874	0.7919	0.165*	
H26C	0.7911	0.2034	0.7699	0.165*	
C27	0.8136 (17)	0.5420 (17)	0.75951 (19)	0.106 (4)	
H27A	0.7483	0.6547	0.7564	0.160*	
H27B	0.8090	0.4736	0.7430	0.160*	
H27C	0.9577	0.5593	0.7649	0.160*	

### Atomic displacement parameters (Å^2^)

**Table:**

	*U*^11^	*U*^22^	*U*^33^	*U*^12^	*U*^13^	*U*^23^
O1	0.061 (3)	0.111 (6)	0.060 (3)	0.030 (4)	−0.011 (3)	−0.001 (4)
O2	0.056 (3)	0.074 (4)	0.052 (3)	0.014 (3)	0.000 (3)	−0.002 (3)
O3	0.053 (3)	0.096 (5)	0.059 (3)	−0.013 (4)	0.001 (3)	−0.006 (4)
O4	0.080 (4)	0.075 (5)	0.085 (5)	−0.023 (4)	0.014 (4)	0.001 (4)
O5	0.099 (5)	0.150 (8)	0.104 (6)	−0.062 (6)	0.009 (4)	−0.035 (5)
O6	0.043 (3)	0.127 (6)	0.087 (4)	0.011 (4)	−0.017 (3)	0.026 (4)
C1	0.041 (4)	0.077 (7)	0.054 (5)	0.008 (5)	0.009 (4)	0.006 (5)
C2	0.038 (4)	0.071 (6)	0.050 (4)	0.014 (4)	−0.004 (3)	−0.006 (4)
C3	0.040 (4)	0.061 (6)	0.050 (4)	0.007 (4)	−0.007 (4)	0.001 (4)
C4	0.051 (4)	0.061 (5)	0.047 (4)	0.002 (4)	−0.003 (4)	0.002 (4)
C5	0.064 (5)	0.060 (6)	0.061 (5)	0.007 (5)	0.000 (4)	0.006 (5)
C6	0.066 (6)	0.107 (10)	0.074 (7)	−0.005 (7)	0.011 (6)	−0.021 (7)
C7	0.068 (5)	0.052 (6)	0.053 (5)	−0.002 (5)	0.004 (4)	0.012 (4)
C8	0.072 (6)	0.081 (7)	0.063 (6)	0.004 (6)	0.014 (5)	0.006 (5)
C9	0.096 (7)	0.088 (8)	0.085 (7)	0.001 (7)	0.037 (6)	0.006 (7)
C10	0.155 (11)	0.080 (8)	0.069 (7)	−0.001 (9)	0.037 (8)	−0.007 (6)
C11	0.154 (10)	0.071 (8)	0.064 (7)	−0.012 (8)	−0.006 (7)	0.001 (6)
C12	0.099 (6)	0.069 (7)	0.058 (5)	−0.004 (6)	−0.001 (5)	0.008 (5)
C13	0.044 (4)	0.076 (7)	0.073 (6)	0.006 (5)	0.007 (4)	−0.009 (5)
C14	0.052 (4)	0.093 (8)	0.074 (6)	−0.003 (5)	0.003 (4)	0.020 (6)
C15	0.050 (4)	0.060 (6)	0.055 (5)	0.006 (4)	0.001 (4)	0.002 (5)
C16	0.076 (6)	0.077 (8)	0.129 (9)	0.033 (6)	0.020 (7)	−0.002 (7)
C17	0.075 (6)	0.098 (9)	0.105 (8)	−0.001 (7)	0.015 (6)	0.014 (7)
C18	0.132 (9)	0.053 (6)	0.065 (6)	0.013 (7)	−0.008 (6)	−0.002 (5)
C19	0.100 (6)	0.066 (7)	0.069 (6)	−0.007 (6)	0.001 (5)	−0.011 (5)
C20	0.122 (8)	0.089 (8)	0.065 (6)	0.020 (8)	0.023 (6)	0.012 (6)
C21	0.105 (8)	0.082 (8)	0.087 (8)	−0.007 (7)	−0.024 (7)	0.002 (6)
C22	0.078 (6)	0.075 (8)	0.145 (10)	0.006 (6)	−0.002 (8)	0.011 (9)
C23	0.087 (7)	0.087 (9)	0.098 (8)	0.001 (7)	0.027 (6)	0.000 (7)
C24	0.070 (5)	0.098 (8)	0.050 (5)	0.005 (6)	−0.003 (4)	−0.012 (6)
C25	0.115 (8)	0.146 (12)	0.092 (7)	−0.013 (9)	−0.019 (7)	−0.051 (8)
C26	0.182 (12)	0.073 (8)	0.075 (7)	0.005 (9)	0.005 (8)	−0.022 (6)
C27	0.128 (8)	0.127 (10)	0.065 (6)	0.014 (9)	0.019 (6)	−0.002 (7)

### Geometric parameters (Å, °)

**Table:**

O1—C1	1.209 (9)	C14—H14B	0.9800
O2—C1	1.327 (10)	C14—H14C	0.9800
O2—C24	1.489 (10)	C15—C16	1.487 (13)
O3—C6	1.341 (12)	C15—H15A	0.9900
O3—C4	1.447 (9)	C15—H15B	0.9900
O4—C6	1.345 (11)	C16—C17	1.286 (8)
O4—C5	1.438 (10)	C16—H16A	0.9500
O5—C6	1.195 (11)	C17—C18	1.548 (12)
O6—C13	1.208 (9)	C17—H17A	0.9500
C1—C2	1.542 (11)	C18—C19	1.3900
C2—C15	1.544 (12)	C18—C23	1.3900
C2—C3	1.550 (11)	C19—C20	1.3900
C2—C13	1.564 (12)	C19—H19A	0.9500
C3—C4	1.476 (10)	C20—C21	1.3900
C3—H3A	0.9900	C20—H20A	0.9500
C3—H3B	0.9900	C21—C22	1.3900
C4—C5	1.539 (11)	C21—H21A	0.9500
C4—H4A	1.0000	C22—C23	1.3900
C5—C7	1.500 (9)	C22—H22A	0.9500
C5—H5A	1.0000	C23—H23A	0.9500
C7—C8	1.3900	C24—C26	1.512 (14)
C7—C12	1.3900	C24—C25	1.515 (14)
C8—C9	1.3900	C24—C27	1.528 (14)
C8—H8A	0.9500	C25—H25A	0.9800
C9—C10	1.3900	C25—H25B	0.9800
C9—H9A	0.9500	C25—H25C	0.9800
C10—C11	1.3900	C26—H26A	0.9800
C10—H10A	0.9500	C26—H26B	0.9800
C11—C12	1.3900	C26—H26C	0.9800
C11—H11A	0.9500	C27—H27A	0.9800
C12—H12A	0.9500	C27—H27B	0.9800
C13—C14	1.511 (11)	C27—H27C	0.9800
C14—H14A	0.9800		
			
C1—O2—C24	121.3 (7)	C13—C14—H14C	109.5
C6—O3—C4	109.6 (6)	H14A—C14—H14C	109.5
C6—O4—C5	110.3 (8)	H14B—C14—H14C	109.5
O1—C1—O2	127.8 (8)	C16—C15—C2	112.0 (6)
O1—C1—C2	122.8 (9)	C16—C15—H15A	109.2
O2—C1—C2	109.4 (7)	C2—C15—H15A	109.2
C1—C2—C15	109.1 (7)	C16—C15—H15B	109.2
C1—C2—C3	112.5 (7)	C2—C15—H15B	109.2
C15—C2—C3	113.0 (6)	H15A—C15—H15B	107.9
C1—C2—C13	107.5 (6)	C17—C16—C15	125.7 (10)
C15—C2—C13	106.7 (8)	C17—C16—H16A	117.2
C3—C2—C13	107.8 (7)	C15—C16—H16A	117.2
C4—C3—C2	115.3 (6)	C16—C17—C18	124.6 (10)
C4—C3—H3A	108.5	C16—C17—H17A	117.7
C2—C3—H3A	108.5	C18—C17—H17A	117.7
C4—C3—H3B	108.5	C19—C18—C23	120.0
C2—C3—H3B	108.5	C19—C18—C17	112.7 (6)
H3A—C3—H3B	107.5	C23—C18—C17	127.3 (6)
O3—C4—C3	111.6 (7)	C20—C19—C18	120.0
O3—C4—C5	102.4 (7)	C20—C19—H19A	120.0
C3—C4—C5	115.2 (6)	C18—C19—H19A	120.0
O3—C4—H4A	109.1	C19—C20—C21	120.0
C3—C4—H4A	109.1	C19—C20—H20A	120.0
C5—C4—H4A	109.1	C21—C20—H20A	120.0
O4—C5—C7	110.1 (6)	C22—C21—C20	120.0
O4—C5—C4	102.0 (6)	C22—C21—H21A	120.0
C7—C5—C4	116.1 (7)	C20—C21—H21A	120.0
O4—C5—H5A	109.4	C21—C22—C23	120.0
C7—C5—H5A	109.4	C21—C22—H22A	120.0
C4—C5—H5A	109.4	C23—C22—H22A	120.0
O5—C6—O3	125.2 (10)	C22—C23—C18	120.0
O5—C6—O4	123.9 (11)	C22—C23—H23A	120.0
O3—C6—O4	110.8 (9)	C18—C23—H23A	120.0
C8—C7—C12	120.0	O2—C24—C26	100.5 (7)
C8—C7—C5	120.4 (5)	O2—C24—C25	109.8 (8)
C12—C7—C5	119.5 (5)	C26—C24—C25	115.6 (13)
C9—C8—C7	120.0	O2—C24—C27	107.6 (8)
C9—C8—H8A	120.0	C26—C24—C27	109.8 (9)
C7—C8—H8A	120.0	C25—C24—C27	112.6 (10)
C8—C9—C10	120.0	C24—C25—H25A	109.5
C8—C9—H9A	120.0	C24—C25—H25B	109.5
C10—C9—H9A	120.0	H25A—C25—H25B	109.5
C11—C10—C9	120.0	C24—C25—H25C	109.5
C11—C10—H10A	120.0	H25A—C25—H25C	109.5
C9—C10—H10A	120.0	H25B—C25—H25C	109.5
C10—C11—C12	120.0	C24—C26—H26A	109.5
C10—C11—H11A	120.0	C24—C26—H26B	109.5
C12—C11—H11A	120.0	H26A—C26—H26B	109.5
C11—C12—C7	120.0	C24—C26—H26C	109.5
C11—C12—H12A	120.0	H26A—C26—H26C	109.5
C7—C12—H12A	120.0	H26B—C26—H26C	109.5
O6—C13—C14	121.3 (7)	C24—C27—H27A	109.5
O6—C13—C2	120.2 (8)	C24—C27—H27B	109.5
C14—C13—C2	118.5 (8)	H27A—C27—H27B	109.5
C13—C14—H14A	109.5	C24—C27—H27C	109.5
C13—C14—H14B	109.5	H27A—C27—H27C	109.5
H14A—C14—H14B	109.5	H27B—C27—H27C	109.5
			
C24—O2—C1—O1	−1.1 (11)	C7—C8—C9—C10	0.0
C24—O2—C1—C2	−179.0 (6)	C8—C9—C10—C11	0.0
O1—C1—C2—C15	6.8 (10)	C9—C10—C11—C12	0.0
O2—C1—C2—C15	−175.2 (6)	C10—C11—C12—C7	0.0
O1—C1—C2—C3	133.0 (8)	C8—C7—C12—C11	0.0
O2—C1—C2—C3	−49.0 (8)	C5—C7—C12—C11	−178.0 (6)
O1—C1—C2—C13	−108.5 (9)	C1—C2—C13—O6	−144.0 (9)
O2—C1—C2—C13	69.5 (9)	C15—C2—C13—O6	99.1 (10)
C1—C2—C3—C4	−63.4 (9)	C3—C2—C13—O6	−22.5 (12)
C15—C2—C3—C4	60.6 (10)	C1—C2—C13—C14	39.9 (12)
C13—C2—C3—C4	178.2 (7)	C15—C2—C13—C14	−77.0 (9)
C6—O3—C4—C3	142.5 (7)	C3—C2—C13—C14	161.4 (7)
C6—O3—C4—C5	18.7 (8)	C1—C2—C15—C16	−172.8 (7)
C2—C3—C4—O3	72.6 (10)	C3—C2—C15—C16	61.3 (9)
C2—C3—C4—C5	−171.2 (7)	C13—C2—C15—C16	−56.9 (9)
C6—O4—C5—C7	141.5 (7)	C2—C15—C16—C17	−108.2 (11)
C6—O4—C5—C4	17.6 (9)	C15—C16—C17—C18	174.1 (8)
O3—C4—C5—O4	−21.1 (7)	C16—C17—C18—C19	−168.3 (10)
C3—C4—C5—O4	−142.4 (8)	C16—C17—C18—C23	10.7 (15)
O3—C4—C5—C7	−140.8 (7)	C23—C18—C19—C20	0.0
C3—C4—C5—C7	97.9 (9)	C17—C18—C19—C20	179.1 (7)
C4—O3—C6—O5	169.6 (10)	C18—C19—C20—C21	0.0
C4—O3—C6—O4	−8.5 (10)	C19—C20—C21—C22	0.0
C5—O4—C6—O5	175.1 (10)	C20—C21—C22—C23	0.0
C5—O4—C6—O3	−6.7 (10)	C21—C22—C23—C18	0.0
O4—C5—C7—C8	−53.3 (8)	C19—C18—C23—C22	0.0
C4—C5—C7—C8	61.9 (8)	C17—C18—C23—C22	−178.9 (8)
O4—C5—C7—C12	124.7 (6)	C1—O2—C24—C26	−174.4 (8)
C4—C5—C7—C12	−120.1 (6)	C1—O2—C24—C25	−52.1 (13)
C12—C7—C8—C9	0.0	C1—O2—C24—C27	70.8 (9)
C5—C7—C8—C9	178.0 (6)		

### Hydrogen-bond geometry (Å, °)

**Table:**

*D*—H···*A*	*D*—H	H···*A*	*D*···*A*	*D*—H···*A*
C19—H19A···Cg	0.95	3.03	3.757	135
